# Expression of glycolytic enzymes in ovarian cancers and evaluation of the glycolytic pathway as a strategy for ovarian cancer treatment

**DOI:** 10.1186/s12885-018-4521-4

**Published:** 2018-06-05

**Authors:** Chrysi Xintaropoulou, Carol Ward, Alan Wise, Suzanna Queckborner, Arran Turnbull, Caroline O. Michie, Alistair R. W. Williams, Tzyvia Rye, Charlie Gourley, Simon P. Langdon

**Affiliations:** 1 Cancer Research UK Edinburgh Centre and Division of Pathology Laboratory, Institute of Genetics and Molecular Medicine, University of Edinburgh, Edinburgh, EH4 2XU UK; 2 The Royal (Dick) School of Veterinary Studies and Roslin Institute, Easter Bush, Roslin, Midlothian, EH25 9RG UK; 3 IOmet Pharma (a wholly owned subsidiary of Merck & Co., Inc., Kenilworth, NJ USA, known as MSD outside the United States and Canada) Nine Edinburgh Bioquarter, Little France Road, Edinburgh, EH16 4UX UK; 4 Cancer Research UK Edinburgh Centre, Institute of Genetics and Molecular Medicine, University of Edinburgh, Edinburgh, EH4 2XU UK; 5 Division of Pathology, University of Edinburgh Medical School, 51 Little France Crescent, Edinburgh, EH16 4SA UK

**Keywords:** Ovarian cancer, Glycolytic pathway, Inhibitors, Combination strategies, Cisplatin, Metformin

## Abstract

**Background:**

Novel therapeutic approaches are required to treat ovarian cancer and dependency on glycolysis may provide new targets for treatment. This study sought to investigate the variation of expression of molecular components (GLUT1, HKII, PKM2, LDHA) of the glycolytic pathway in ovarian cancers and the effectiveness of targeting this pathway in ovarian cancer cell lines with inhibitors.

**Methods:**

Expression of GLUT1, HKII, PKM2, LDHA were analysed by quantitative immunofluorescence in a tissue microarray (TMA) analysis of 380 ovarian cancers and associations with clinicopathological features were sought. The effect of glycolysis pathway inhibitors on the growth of a panel of ovarian cancer cell lines was assessed by use of the SRB proliferation assay. Combination studies were undertaken combining these inhibitors with cytotoxic agents.

**Results:**

Mean expression levels of GLUT1 and HKII were higher in high grade serous ovarian cancer (HGSOC), the most frequently occurring subtype, than in non-HGSOC. GLUT1 expression was also significantly higher in advanced stage (III/IV) ovarian cancer than early stage (I/II) disease. Growth dependency of ovarian cancer cells on glucose was demonstrated in a panel of ovarian cancer cell lines. Inhibitors of the glycolytic pathway (STF31, IOM-1190, 3PO and oxamic acid) attenuated cell proliferation in platinum-sensitive and platinum-resistant HGSOC cell line models in a concentration dependent manner. In combination with either cisplatin or paclitaxel, 3PO (a novel PFKFB3 inhibitor) enhanced the cytotoxic effect in both platinum sensitive and platinum resistant ovarian cancer cells. Furthermore, synergy was identified between STF31 (a novel GLUT1 inhibitor) or oxamic acid (an LDH inhibitor) when combined with metformin, an inhibitor of oxidative phosphorylation, resulting in marked inhibition of ovarian cancer cell growth.

**Conclusions:**

The findings of this study provide further support for targeting the glycolytic pathway in ovarian cancer and several useful combinations were identified.

**Electronic supplementary material:**

The online version of this article (10.1186/s12885-018-4521-4) contains supplementary material, which is available to authorized users.

## Background

Ovarian cancer is the 7th most common female cancer worldwide with an estimated 239,000 new diagnoses worldwide each year [1]. Standard treatment of ovarian cancer consists of debulking surgery followed by systemic platinum and taxane-based chemotherapy. Even though platinum-based chemotherapy has a high response rate, it is estimated that approximately 70% of patients will relapse with resistant disease and new treatments are required [2]. High-grade serous ovarian cancer (HGSOC) accounts for approximately 70% of epithelial ovarian cancers while non-HGSOC which includes endometrioid, clear cell, mucinous and low-grade serous ovarian cancer, among others, comprise important subgroups [2].

Many cancer cells rely on glycolysis as their primary source of energy regardless of oxygen availability; the persistence of glycolysis in cancer cells even under aerobic conditions is termed aerobic glycolysis or the Warburg effect. This metabolic alteration in tumours has been extensively demonstrated in a wide variety of cancers and considered a ‘hallmark’ of advanced malignancy [3–5]. It has been estimated that many tumour cells under aerobic conditions produce up to 60% of their ATP requirement through glycolysis [6, 7]. This ‘metabolic reprogramming’ is an adaptation to meet the requirements of highly proliferative malignant tissues, providing the precursors needed to support biosynthesis [8, 9]. Furthermore, the metabolic alteration of cancer cells can provide them with a selective advantage for survival and growth in low oxygen tumour microenvironments. As tumours grow and expand away from a functional blood supply, glycolysis is an evolutionary adaptation of cells to survive and thrive in a hypoxic environment [3, 7, 10]. This reliance on glycolysis provides a possible therapeutic opportunity and the enzymes comprising the glycolytic pathway may be potential targets for cancer treatment [6, 10–17]. Several glycolytic inhibitors have emerged as exhibiting promising anticancer activity both in vitro and in vivo and a number have reached clinical trials [10–13, 16].

Glucose transporter 1 (GLUT1) is the first component of the glycolysis pathway, transporting glucose into the cell, and is up-regulated in many tumour types. High expression has been associated with poor clinical outcome and adverse prognosis [18–20]. STF31 [4-[[[[4-(1, 1-Dimethylethyl) phenyl] sulfonyl] amino] methyl]-N-3-pyridinylbenzamide] is a pyridyl-anilino-thiazole that impairs glycolytic metabolism and binds to the GLUT1 transporter [21]. Based on molecular modelling, STF31 was predicted to interact directly with the central pore of the transporter and was shown to inhibit glucose uptake and induce necrotic cell death selectively in glycolytic cancer cells. In vivo efficacy of the compound was also demonstrated [21]. IOM-1190 is a GLUT1 inhibitor that suppresses 2-deoxy-D-glucose (2-DG) uptake and lactate production in A549 lung cancer cells resulting in rapid apoptotic cell death. High affinity for GLUT1 binding of the radiolabelled compound has also been documented [22].

Hexokinase catalyses the first rate-controlling irreversible reaction of the glycolytic pathway; phosphorylating glucose to glucose-6-phosphate coupled with ATP de-phosphorylation. The mitochondrial-bound isoform HKII is considered to play a pivotal role in carcinogenesis and is overexpressed in many tumours [23, 24].

6-Phosphofructo-2-kinase/fructose-2,6-biphosphatase (3PFKFB3), which converts fructose-6-phosphate to fructose-2,6-bisP (F2,6BP), is downstream of HKII. PFKFB3 overexpression has been documented in several tumour types including ovarian cancers [25]. In 2008, Clem et al. identified a competitive inhibitor of PFKFB3, 3PO, using computational modelling and virtual database in silico screening. 3PO [3-(3-Pyridinyl)-1-(4-pyridinyl)-2-propen-1-one] is a novel small molecule, dipyridinyl-propenone based compound that reduced intracellular F2,6BP levels, glucose uptake and lactate production followed by induction of G2-M phase cell cycle arrest. 3PO treatment suppressed tumour growth in vivo in mice bearing leukaemia, lung and breast adenocarcinoma xenografts [26].

Further downstream is the M2 isozyme of pyruvate kinase (PKM2) which catalyses the irreversible conversion of phosphoenolpyruvate (PEP) to pyruvate coupled with ADP phosphorylation and is found overexpressed in various tumour types and plays a pivotal role in carcinogenesis [27, 28].

Lactate dehydrogenase A (LDHA) is the enzyme catalysing the reduction of pyruvate in the final step of the glycolytic pathway. LDHA upregulation has been reported in ovarian cancers when compared to normal tissues [29]. LDHA overexpression is considered to have a crucial role in tumorigenesis and is often associated with poor clinical outcome and resistance to therapy [30–32]. Oxamic acid is an established pyruvate analogue (a structural isostere of pyruvic acid) described as a well characterised substrate-like competitive inhibitor of LDH. Promising anti-proliferative effects of oxamic acid have been reported in vitro in hepatocellular and breast carcinoma cell lines [33–36].

Several successful combinations of glycolytic inhibitors with cytotoxic drugs have recently been identified and glycolytic inhibitors have been demonstrated to resensitise drug-resistant cells to conventional regimens [12, 14, 15, 37–39].

We have previously demonstrated antitumour activity of glycolytic inhibitors against panels of ovarian and breast cancer cell lines [40]. In the present study, we evaluated the levels of expression of four selected glycolytic targets (GLUT1, HKII, PKM2 and LDHA) in a large series of ovarian cancers to investigate possible associations with histological subtype and stage of disease. We have then used four inhibitors to target prime components of the pathway and compared these agents against paired chemosensitive and chemoresistant ovarian cancer cell lines. Novel combinations between cisplatin and paclitaxel with inhibitors of the glycolytic pathway were then investigated and evaluated quantitatively by comparison of their combination indices.

## Methods

### Study population

Primary Ovarian cancer patients treated at the Edinburgh Cancer Centre between 1991 and 2006 were retrospectively identified from the Edinburgh Ovarian Cancer Database. Tissues were formalin-fixed and paraffin-embedded. Haematoxylin-eosin stained slides were reviewed by a subspecialist gynaecological pathologist, and histological classification of tumour type confirmed. Three separate Tissue Microarray (TMA) replicates containing cores of 380 ovarian tumours were constructed. The number of samples available for histology and stage analysis is shown in Additional file 1: Table S1 and the full dataset used for analysis is given in Additional file 2.

No informed consent was obtained for use of retrospective tissue samples from the patients within this study, most of whom were deceased, since this was not deemed necessary by the Ethics Committee. The TMA material was kindly provided by the Edinburgh Experimental Cancer Medicine Centre (ECMC ID: SR319). Ethical approval for the use of tumour material and correlation with associated clinical data was obtained from South East Scotland Human Annotated Bioresource (East of Scotland Research Ethics Service Reference 15/ES/0094).

### Immunofluorescence of clinical ovarian cancer tissues

Microscope slides of TMA sections were deparaffinised and rehydrated followed by heat-induced antigen retrieval being performed in sodium citrate buffer at pH 6. Endogenous peroxidase activity was blocked with 3% hydrogen peroxide for 10 min and non-specific binding was blocked by a 10 min incubation in serum-free protein block (DAKO). Primary antibodies were diluted in antibody diluent (DAKO) and were applied overnight at 4 °C. The following primary rabbit antibodies, validated for the protocol, were used: GLUT1 (Merck Millipore), HKII (Cell Signaling Technology), LDHA (Cell Signaling Technology) and PKM2 (Cell Signaling Technology). The following day, tissue sections were washed with 0.05% PBS Tween 20 (PBS-T), and were then incubated with primary mouse anti-cytokeratin antibody (M3515/DAKO) diluted 1:25 in the same antibody diluent in order to mask the tumour areas. This incubation was performed at room temperature, lasted 1 h and was followed by PBS-T washes. To enable epithelial mask visualisation, slides were then incubated with the secondary goat anti-mouse antibody conjugated with Alexa Fluor 555 (Thermo Fisher Scientific) diluted 1:25 in the goat anti-rabbit peroxidase-conjugated Envision reagent (DAKO). This incubation was conducted at room temperature protected from light for 90 min and was followed by PBS-T washes. Target visualisation was implemented by a 10 min incubation with Cyanine 5 (Cy5) Tyramide, diluted at 1:50 in amplification diluent (PerkinElmer), at room temperature protected from light. Subsequently, tissue sections were washed with PBS-T and dehydrated. Finally, slides were counterstained with 45 μl Prolong Gold Antifade Mountant with DAPI (4′, 6-diamidino-2-phenylindole) (Thermo Fisher Scientific) to visualise the nuclei and a coverslip was mounted.

### AQUA image analysis

Protein expression in the ovarian tumour cores was quantitatively evaluated by Automated Quantitative Analysis (AQUA) [41]. High resolution monochromatic images of each TMA core were captured at 20× objective using an Olympus AX-51 epifluorescence microscope and were analysed by AQUAnalysis software. DAPI, Cy-3 and Cy-5 filters were applied to visualise the nuclei, the cytokeratin tumour mask and the target protein respectively. The Cy-5 fluorescent signal intensity of the target antigen was quantified in each image pixel. A quantitative score was attributed to each histospot based on the average Cy5 signal in the cytoplasmic compartment within the epithelial tumour mask, as identified by the cytokeratin Cy3 stain. Damaged cores or cores containing imaging errors as well as those consisting of less than 5% epithelium were excluded from further analysis.

Target expression in the cytoplasmic compartment of each core was quantified and assigned an AQUA score. Data were filtered and only samples that had at least two replicate values were considered. Expression values were averaged from either two or three replicates. Spearman non-parametric correlation and network analysis were conducted using TMA Navigator [42]. Correlation heatmaps were generated using the same software (http://www.tmanavigator.org/). For this analysis, expression data of different markers had been log2 transformed, mean-centred and quantile-normalised to compensate for differences in the staining. The expression of examined glycolytic targets was compared across the different pathological stages and histological types of ovarian tumours using one-way ANOVA and statistical significance was determined by the Tukey’s multiple comparisons test. The Spearman correlation coefficient was calculated for each pair of markers and statistical significance was determined using the Algorithm AS89 [43]. Spearman’s correlation *P*-values were adjusted for multiple hypothesis testing according to Benjamini-Yekutieli FDR correction. The P-value significance threshold was set at 0.01.

### Cell lines

A panel of four ovarian cancer cell lines were used initially. OVCAR5, OVCAR3 and CAOV3 are HGSOC cell lines [44] while TOV112D is of endometrioid ovarian cancer origin [45]. OVCAR5 and OVCAR3 were gifts from Dr. Tom Hamilton, Fox Chase Institute, Philadelphia, PA USA while CAOV3 and TOV112D were obtained from American Type Culture Collection, Manassas, Virginia, USA. Two cell line pairs derived from two patients with HGSOC at different stages of platinum-based chemotherapy were also used – PEA1 / PEA2 and PE01/PE04 respectively [46]. The first cell line of each pair was regarded as chemosensitive and the second cell line (which was isolated following the development of platinum resistance), chemoresistant [46, 47]. These were developed within our laboratory and are now available at the European Collection of Cell Cultures, Porton Down, UK. All cell lines used in this study were authenticated using Short Tandem Repeat profiling (STR) (by ECACC) and were routinely subjected to mycoplasma testing.

### Cell culture

All cell line work was conducted in sterile conditions in a class II Laminar Air Flow hood at room temperature. Cells were incubated in a humidified atmosphere of 5% CO2 at 37 °C. The panel of four ovarian cancer cell lines (OVCAR5, TOV112D, OVCAR3 and CAOV3) were all maintained in Dulbecco’s Modified Eagle Medium without HEPES modification (DMEM, Thermo Fisher Scientific), containing glucose (5.56 mM), Sodium Pyruvate (1 mM) and L-glutamine (3.97 mM). The two ovarian cancer cell line pairs (PEA1-PEA2, PEO1-PEO4) were maintained in RPMI 1640 (Thermo Fisher Scientific) containing 11.11 mM glucose and 2 mM L-glutamine. In both cases the media contained phenol red and were supplemented with 10% heat inactivated fetal bovine serum FBS (Fetal Bovine Serum, Thermo Fisher Scientific) and 1% Penicillin-Streptomycin (Penicillin-Streptomycin 10,000 U/mL, Thermo Fisher Scientific).

In the deprivation experiments where the effect of glucose availability on cell growth of different cell lines was examined, medium without glucose was used (DMEM, Thermo Fisher Scientific). Phenol red free media were supplemented with 10% heat inactivated dialysed fetal bovine serum (Thermo Fisher Scientific) and 1% Penicillin-Streptomycin. In the glucose depleted medium the desired concentration of D-Glucose (Sigma Aldrich) was added along with a standard 4 mM L-Glutamine (Sigma Aldrich) concentration.

Cells were routinely maintained in T175cm^3^ tissue culture flasks and were sub-cultured at least once a week, when reaching 70–80% confluence as described below. Medium was discarded and cells were washed with preheated phosphate buffered saline. Cells were then incubated for a few minutes with a trypsin/EDTA solution (Trypsin-EDTA 0.05%, Thermo Fisher Scientific) to cause cell detachment and cell suspension was centrifuged at 1200 rpm for 5 min. Pelleted cells were resuspended in fresh media and transferred into new flasks. When setting up an experiment cells were counted using a Neubauer hemocytometer and were seeded in cell culture plates or dishes at the desired dilution.

### Sulphorhodamine B assay (SRB)

The SRB assay is a colorimetric cell density assay based on the quantification of cellular protein content [48]. Cells were seeded in flat-bottom 96-well plates. After 48 h incubation, cells were treated with or without the relevant treatment as indicated. STF31 and metformin were obtained from Tocris Bioscience, 3PO from Merck Millipore and oxamic acid from Sigma Aldrich. IOM-1190 was provided by IOmet Pharma. The compound is example 187 in patent WO2014/187922 and has an imidazo pyrazine core (https://patents.google.com/patent/WO2014187922A1/en).

Cisplatin (Teva UK Limited) and paclitaxel (Actavis) were obtained as formulated drugs. Stock solutions of compounds were prepared in DMSO except for oxamic acid and metformin which were dissolved in PBS. A series of 10 dilutions with 1:2 steps of each inhibitor in six replicates was applied. Once the treatment period was completed, cell monolayers were fixed on the day of treatment (Day 0 control) and on selected time points thereafter with cold 25% trichloroacetic acid (TCA, Sigma Aldrich). Then cell monolayers were stained with 0.4% SRB dye solution (Sigma Aldrich) and unbound excess dye was removed by 1% glacial acetic acid (VWR International) washes. The protein bound stain was solubilised in 10 mM Tris buffer solution pH 10.5 (Sigma Aldrich). Finally absorbance was measured at 540 nm using a plate reader.

Measurements were corrected for background absorbance and values are presented as percentage of absorbance of untreated control. The half maximal inhibitory concentration (IC_50_), indicating the concentration needed to reduce cell viability by half, was used as a quantitative indication of the effectiveness of each compound as a cancer cell growth inhibitor. IC_50_ values were generated through sigmoidal concentration response curves fitted using the XL fit tool within Microsoft Excel.

### Combinatorial treatments

In combination drug studies, glycolytic inhibitors were assessed in combination with traditional drugs. For these treatments a range of different concentrations of the glycolytic inhibitor were combined with a constant fixed concentration, around the IC_20_ or less, of the other drug. Both drugs were delivered at the same time and cancer cell proliferation was examined by the SRB assay after a 3-day treatment period. Concentration response curves of each examined combination along with curves of the two compounds as single agents were analysed using Calcusyn Software (Biosoft). To quantitatively evaluate the effectiveness of each combination, CI values were generated for each combination point indicating synergy, additivity or antagonism [49]. CI values lower than 0.8 indicate synergy, values between 0.8 and 1.2 imply additivity while values higher than 1.2 indicate antagonism [49].

### Statistical analysis

Statistical tests were undertaken using GraphPad Prism software version 6. Student’s t-test was used to compare two groups and ANOVA followed by the Tukey post-test was used to compare more than two groups. For survival analysis, we undertook Kaplan Meier analysis using X-tile [50] which allows determination of the minimal *p*-value using the Miller-Siegmund minimal P correction.

## Results

### Expression of glycolytic enzymes in ovarian tumours and association with histological subtypes and stage

**Fig. 1:**
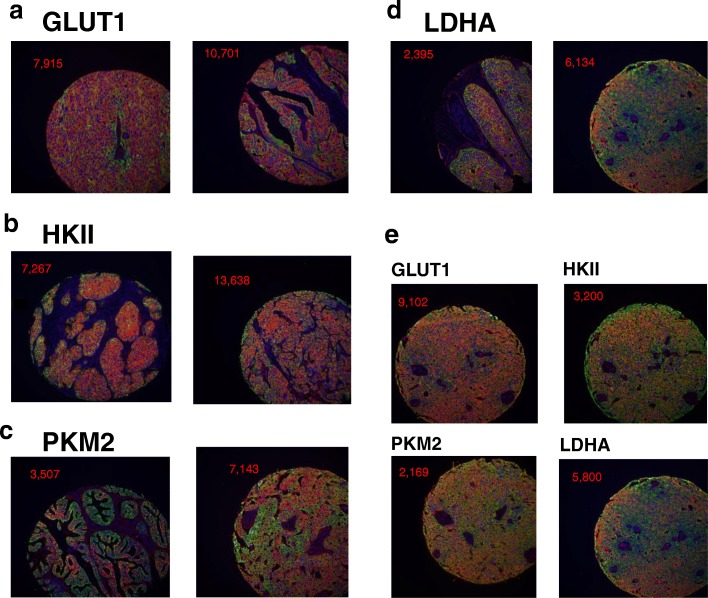
**a**-**d**. Representative immunofluorescence images showing GLUT1, HKII, PKM2 and LDHA expression in TMA cores of ovarian cancers. **e**. Immunofluorescence images showing expression of four glycolytic enzymes in TMA cores of an individual ovarian cancer patient. Blue colour visualises DAPI nuclear counterstain, green colour cytokeratin tumour mask and red colour target staining. Quantified target expression (AQUA value) in the cytoplasmic compartment of each core is indicated

To assess the variation in expression of key components of the glycolytic pathway in ovarian cancers, expression levels of GLUT1, HKII, PKM2 and LDHA were investigated in a series of 380 ovarian tumours by Automated Quantitative Analysis (AQUA). A three label immunofluorescent protocol was used generating a quantitative score for each tumour core. Representative immunofluorescence images illustrating the expression of the four glycolytic targets in TMA cores of ovarian cancers are shown in Fig. 1a-d. GLUT1 showed membrane as well as cytoplasmic localisation while HKII, PKM2 and LDHA demonstrated cytoplasmic localisation (Fig. 1a-d). In Fig. 1e, the expression of the four proteins is shown for an individual ovarian cancer case illustrating high expression for all four consistent with a glycolytic phenotype.

**Fig. 2:**
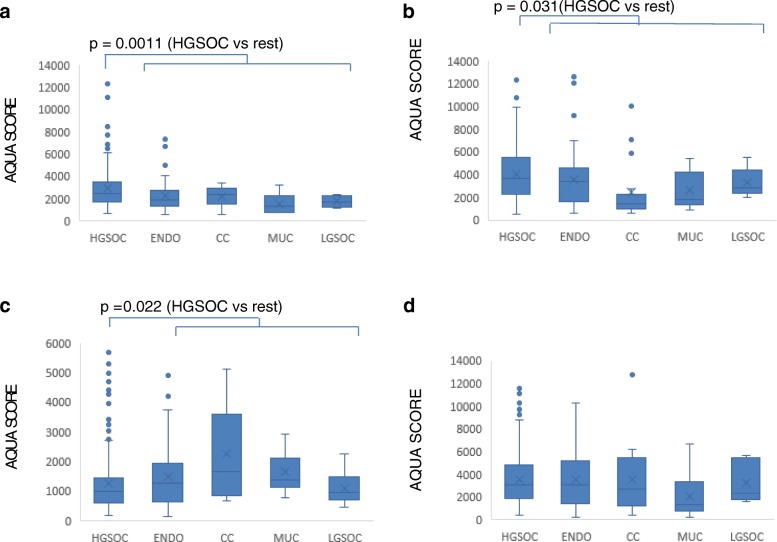
Expression levels of four glycolytic enzymes in different histological subtypes of ovarian cancer. AQUA levels of **a**) GLUT1, **b**) HKII, **c**) LDHA and **d**) PKM2 are shown. Values were measured as described in Methods section. The boxplot shows the median value, with the rectangle representing the 2nd and 3rd quartiles. Statistical significance indicated (Student’s t-test)

Associations between the level of expression of the four molecules and the histological subtype of ovarian cancer were then examined (Fig. 2). High-grade serous ovarian cancer (HGSOC) accounts for approximately 70% of epithelial ovarian cancers [2] and was first compared with non-HGSOC disease. Mean expression of GLUT1 was higher in HGSOC than in non-HGSOC samples (*P* = 0.0011; t-test) (Fig. 2a). Similarly, HKII expression was higher in HGSOC than non-HGSOC (*P* = 0.031; t-test) and this was reflected in a difference between HGSOC and clear cell disease (*P* < 0.05; Tukey test post ANOVA) (Fig. 2b). In contrast, LDHA expression was lower in HGSOC than in non-HGSOC (*P* = 0.022; t-test) and again this difference was reflected in HGSOC being lower than clear cell (*P* < 0.01; Tukey test post ANOVA) (Fig. 2c). For PKM2, there were no statistically significant differences between the histological subtypes (Fig. 2d).

**Fig. 3:**
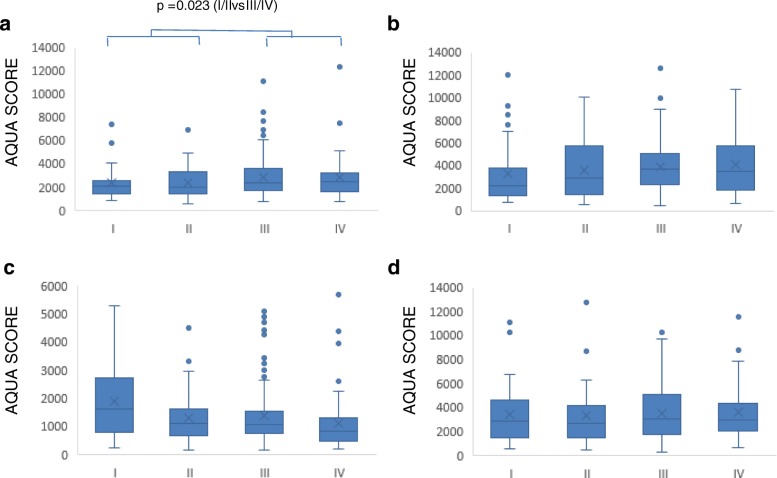
Expression levels of four glycolytic enzymes in different stages of ovarian cancer. AQUA levels of **a**) GLUT1, **b**) HKII, **c**) LDHA and **d**) PKM2 are shown. Values were measured as described in Methods section. The boxplot shows the median value, with the rectangle representing the 2nd and 3rd quartiles

When stage of disease was analysed, GLUT1 expression was higher in advanced disease (stages III/IV) than early disease (stages I/II) (*P* = 0.023; t-test) (Fig. 3a). In contrast, LDHA expression was lower in Stage IV than stage I disease (*P* < 0.05; Tukey test post ANOVA) (Fig. 3c) while no obvious differences emerged for HKII or PKM2. Analysis of the HGSOC group alone indicated no differences in expression between advanced and early stage HGSOC (data not shown). Analysis of patient survival using x-Tile optimal cut-point analysis [50] showed no significant differences in survival with varying expression levels of the four molecules in any of the HGSOC, endometrioid or clear cell cancer groups (data not shown).

**Fig. 4:**
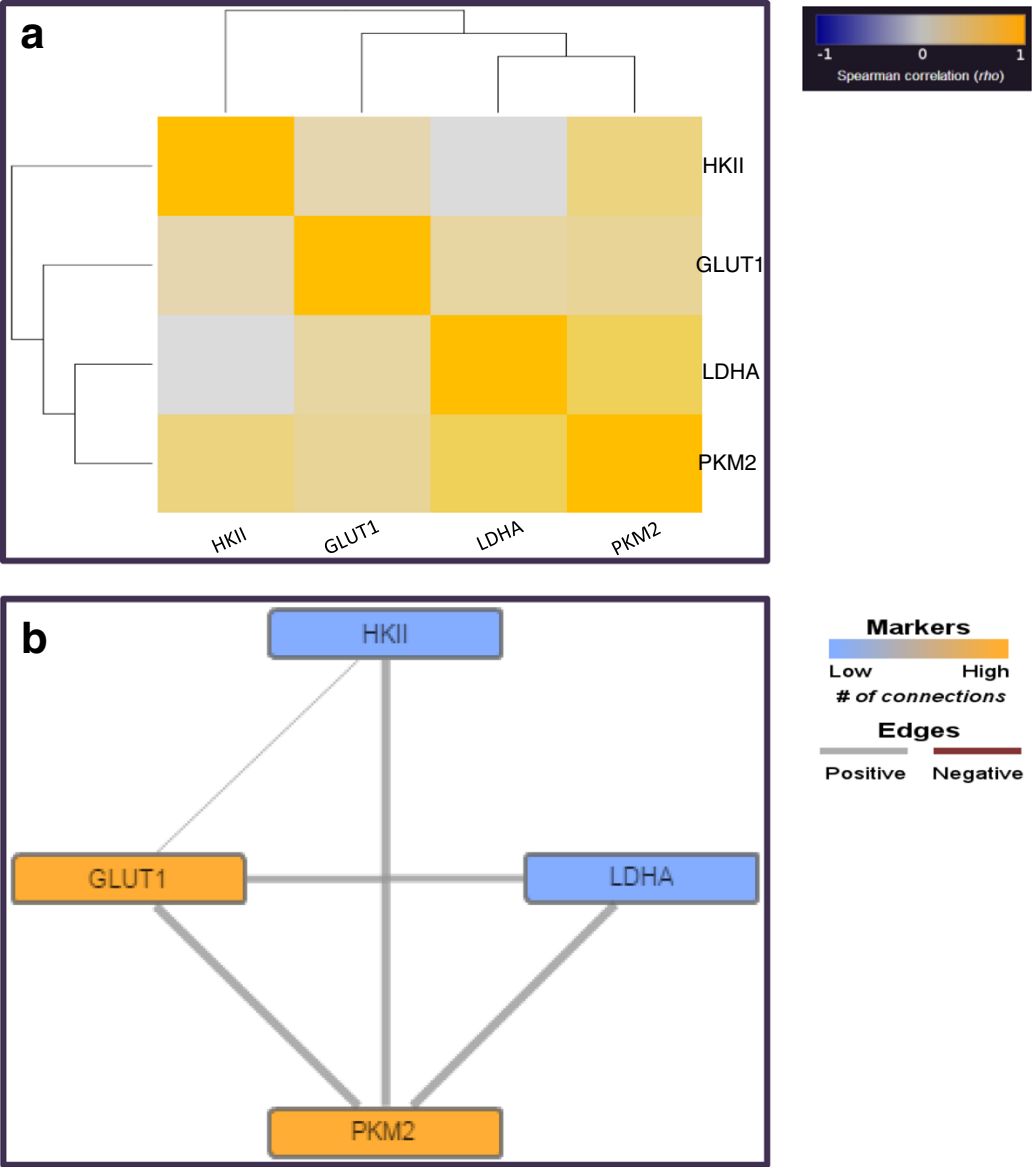
Heatmap and correlation network analysis of the expression of four glycolytic enzymes in a cohort of 380 ovarian cancers. **a**. Heatmap showing the positive Spearman rho correlation values displayed in bright yellow colours and the negative Spearman rho correlation values in dark blue colours. The heatmap was generated using TMA Navigator [42]. **b**: Spearman correlation network of the four glycolytic enzymes in the cohort. Statistically significant correlations thresholded at FDR *P* < 0.01 are presented. High number of significant connections is displayed in bright yellow colours while low in dark blue colours. Positive relationships are indicated in grey while negative in red. Thickness of connection lines reflects significance (the adjusted *P* value). The network was generated using TMA Navigator [42]

A heatmap correlating the expression of the four examined glycolytic enzymes across the dataset is shown in Fig. 4a. Spearman non-parametric correlation was performed and the correlation heatmap was generated using TMA Navigator [43]. The expression of the four targets across the ovarian cancers gave positive rho correlation values when compared to each other. Based on the dendrogram, LDHA expression appeared more closely correlated with PKM2 expression; in contrast HKII expression was more distant to the expression of the other three markers. Spearman correlation network analysis was conducted to further interpret the relationship between the glycolytic markers and evaluate their associations. The correlation network of expression of the four glycolytic enzymes is presented in Fig. 4b. Significant relationships (FDR *P* < 0.01) are drawn as lines that connect pairs of markers. Thickness of connection lines reflects significance and positive significant relationships are displayed in grey colour. The colour of each marker indicates the number of significant connections. High number of significant connections is displayed in yellow colour while low in blue. The correlation values (FDR *P* < 0.01) are summarised in Additional file 3: Table *S*2.

### The effect of glucose on cell growth of a panel of ovarian cancer cell lines

**Fig. 5:**
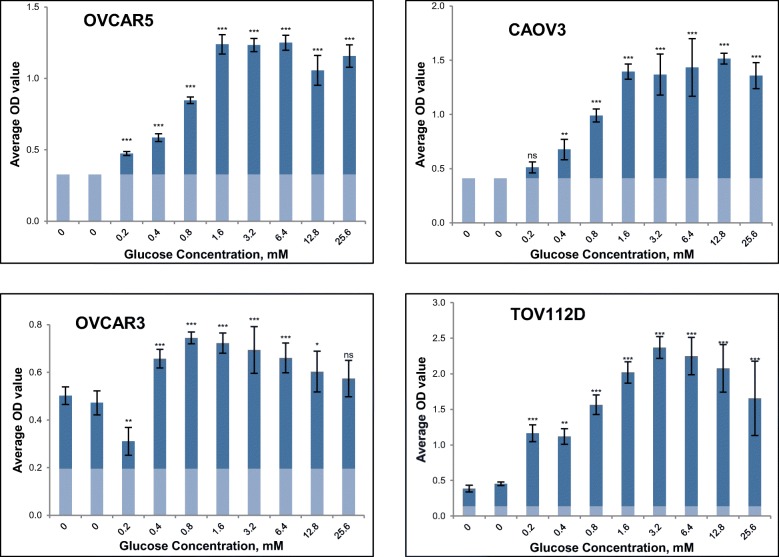
Growth response of a panel of four ovarian cancer cell lines in the presence of varying concentrations of glucose. Glucose concentrations between 0 and 25.6mΜ were evaluated and cells grown for a 5-day period. Optical density was determined by an SRB assay. Mean results of 6 replicates are reported and error bars represent standard deviations. Faint coloration at the bottom of the columns represents OD value on the day of treatment (Day 0). Statistical significance indications: ns not significant *P* > 0.05, * *P* < 0.05, ** P < 0.01, *** *P* < 0.001 compared with the mean of the depleted controls (one-way ANOVA followed by Tukey-Kramer multiple comparisons test)

To assess the growth dependence of ovarian cancer cells on glucose, the proliferation of a small panel of ovarian cancer cell lines was monitored under a range of glucose concentrations after a 5-day incubation period. Growth was compared with controls in medium without glucose. Fig. 5 illustrates the average optical density value generated via SRB assay (indicative of cell number) against increasing concentration of glucose. OVCAR5, CAOV3 and OVCAR3 are of HGSOC origin [45] while TOV112D is of endometrioid cancer origin [46]. OVCAR5 and CAOV3 cells were unable to proliferate when cultured in the absence of glucose for five days; 0.2 mM of glucose was required for significant growth of OVCAR5 cells with higher concentrations leading to higher growth rate until a plateau was reached at 1.6 mM glucose. CAOV3 cells demonstrated significant growth, in comparison to the control samples, when cultured in a minimum of 0.4 mM glucose. In contrast, OVCAR3 and TOV211D cells showed a threefold increase in their cell number in the absence of glucose however were still able to grow more rapidly in the presence of added glucose (Fig. 5).

### The effect of glycolytic inhibitors on cell growth of chemosensitive and chemoresistant HGSOC ovarian cancer cell lines

**Fig. 6:**
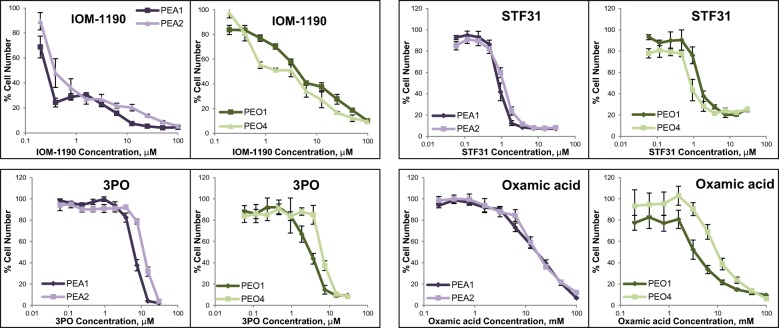
Growth response curves of ovarian cancer cell line pairs treated with glycolysis inhibitors. IOM-1190 was used at concentrations between 0.2-100μΜ, STF31 and 3PO at concentrations between 0.06-30μΜ and oxamic acid at concentrations between 0.4-100mΜ for a 4-day period. Cell viability was determined by an SRB assay. Mean results of 6 replicates are reported and error bars represent standard deviations. Values are shown as a percentage of control. A constant 1% DMSO concentration was used across the whole curve for IOM-1190 and a respective constant 0.3% DMSO concentration for STF31 and 3PO. IC_50_ concentrations are listed in Table 1

PEA1 / PEA2 and PEO1 / PEO4 are two pairs of cancer cell lines established from two individual patients with HGSOC [47]. The first cell line of each pair is platinum sensitive (PEA1 and PE01 respectively) while the second line (PEA2 and PE04 respectively) was acquired after platinum resistance had developed within the patient [47, 48]. Four glycolytic inhibitors (IOM-1190, STF31, 3PO and oxamic acid) were investigated against these ovarian cancer cell line pairs (Fig. 6) and IC_50_ concentrations are listed in Table 1. These inhibitors were selected based on interest in targeting GLUT1 at the top and LDHA at the bottom of the pathway and also on preliminary evidence that the PFKFB3 inhibitor, 3PO, had interesting combinatorial activity in pilot experiments.

**Table 1:** IC_50_ concentrations for glycolysis inhibitors against the PEA1/PEA2 and PE01/PE04 pairs of HGSOC cell lines

IC50 values	1st pair		2nd pair	
	PEA1	PEA2	PEO1	PEO4
IOM-1190 (μM)	0.28	0.46	4.8	1.6
STF31 (μM)	0.86	1.3	1.5	0.88
3PO (μM)	6.3	11.9	3	6.8
Oxamic acid (mM)	16	17.6	3.8	10.1

IOM-1190 is a novel specific GLUT1 inhibitor [22] and attenuated cell proliferation of both chemosensitive and chemoresistant cell lines. PEA1 had an IC_50_ value equal to 280 nM and PEA2 equal to 460 nM. In contrast, the PEO4 platinum-resistant cell line presented greater sensitivity having a threefold lower IC_50_ value (equal to 1.6μΜ) compared to the platinum sensitive PEO1 cell line (4.8 μΜ). STF31, another GLUT1 inhibitor [21] had similar inhibitory activity against both cell lines of each pair. Although also reported as an NAMPT inhibitor [51], it reassuringly had a pattern of activity similar to that of IOM-1190. The PEA2 cell line was slightly more resistant to STF31 compared to its paired platinum naïve line PEA1, with IC_50_ values of 1.3μΜ and 0.9μΜ respectively. In contrast, the platinum-resistant line PEO4, having an IC_50_ value of 0.9μΜ, showed increased sensitivity to the inhibitor compared to its paired platinum-sensitive line PEO1, with an IC_50_ value of 1.5μΜ. 3PO is a recently identified PFKFB3 inhibitor [27]. Sensitivity to 3PO coincided with platinum sensitivity. Both platinum resistant cell lines (PEA2 and PE04) presented greater resistance to 3PO compared to their platinum sensitive paired cell lines with twofold higher IC_50_ value. Oxamic acid is an established LDH inhibitor [34–37]. The first ovarian cancer cell line pair responded similarly to this agent with an almost identical IC_50_ value of 16 mM. Regarding the second pair, the PEO4 platinum resistant cell line proved to be more resistant to oxamic acid, having an IC_50_ value threefold higher than the corresponding value of PEO1 (Table 1). These results indicate that, in general, platinum-resistant disease has comparable sensitivity to these glycolysis inhibitors when compared to chemo-sensitive disease.

### The PFKFB3 inhibitor, 3PO, potentiated the antiproliferative effect of cisplatin and paclitaxel in ovarian cancer cells

**Fig. 7:**
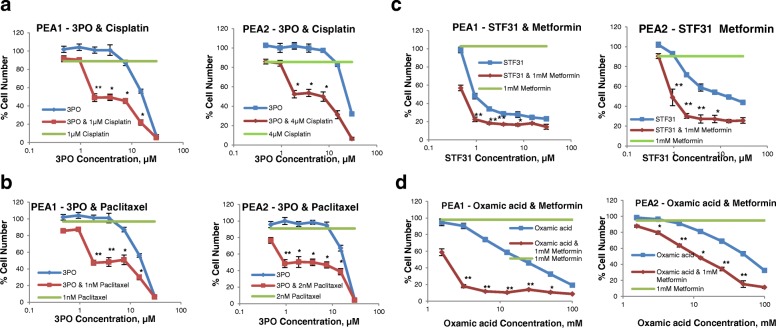
Growth response curves of PEA1 and PEA2 ovarian cancer cells treated with combinations of glycolysis inhibitors with chemotherapy or metformin. **a.** 3PO with cisplatin. 3PO concentrations between 0.5-30μΜ alone (blue line) or combined with a constant concentration of cisplatin (red line) were evaluated. In green the effect of 1μΜ (PEA1) or 4μΜ (PEA2) cisplatin on cell viability is presented. **b**. 3PO with paclitaxel. 3PO concentrations between 0.5-30μΜ alone (blue line) or combined with a constant concentration of paclitaxel (red line) were evaluated. In green the effect of 1μΜ (PEA1) or 2μΜ (PEA2) paclitaxel on cell viability is presented. **c**. STF31 with metformin. Concentration response curves of PEA1 and PEA2 ovarian cancer cells treated with STF31 concentrations between 0.5-30μΜ alone (blue line) or combined with 1 mM metformin (red line). In green the effect of 1 mM metformin on cell viability is presented. **d**. Oxamic acid with metformin. Concentration response curves of PEA1 and PEA2 ovarian cancer cells treated with oxamic acid concentrations between 1.56-100mΜ alone (blue line) or combined with 1 mM metformin (red line). In green the effect of 1 mM metformin on cell viability is presented. Cell viability was determined by an SRB assay after a 3-day treatment. Mean results of 6 replicates are reported and error bars represent standard deviations. Values are shown as a percentage of control. Asterisks indicate synergistic combination points with *CI value lower than 0.8 and **CI value lower than 0.3

Combinations of the PFKFB3 inhibitor, 3PO, with cisplatin and paclitaxel were next investigated against the paired cell lines. 3PO was able to enhance the effect of cisplatin in both the chemosensitive PEA1 and chemoresistant PEA2 cell lines. A range of different concentrations of 3PO were used in combination with a constant fixed concentration (around the IC_20_), of the cytotoxic drug; hence in PEA2 cells, 4μΜ of cisplatin was required to produce a similar inhibitory effect in cell number to that of 1μΜ cisplatin on PEA1 cells. Both drugs were delivered at the same time and cancer cell proliferation was examined by the SRB assay after a 3-day treatment period. Combination Index values (CI) were generated for each combination point, using Calcusyn software, providing a quantitative evaluation of the combination efficacy. Concentrations at which synergistic interactions (CI values lower than 0.8) between the two compounds were identified are indicated by asterisks in Fig. 7a. The combination of 3PO with paclitaxel was also effective in inhibiting growth of the PEA1 and PEA2 cell lines, generating low CI values for all 3PO concentrations used (Fig. 7b). These drug combinations were similarly effective for the other examined ovarian cancer cell line pair PEO1 and PEO4 and also demonstrated synergistic activity (Additional file 4: Fig. S1).

### Metformin potentiated the antiproliferative effect of glycolytic inhibitors on ovarian cancer cells

We have previously reported promising combinatorial activity between metformin and STF31 or oxamic acid in a breast cancer cell line [40]. Metformin inhibits the mitochondrial respiratory chain complex I and combination with a glycolytic inhibitor will result in more complete depletion of cellular ATP. The effect of either STF31 or oxamic acid on both chemosensitive and chemoresistant ovarian cancer cell lines was markedly enhanced by metformin (Fig. 7c). Strong synergy at the level of a CI value equal to 0.1 was demonstrated for both cell lines. These drug interactions were similarly effective for the other examined ovarian cancer cell line pair (PEO1-PEO4, Additional file 4: Fig. S1C).

## Discussion

There is continued interest in the potential of targeting the glycolytic pathway as a therapeutic strategy for cancer treatment [15, 17, 45, 46]. In this study we evaluated the relative expression of several glycolytic markers across a large cohort of clinical ovarian tumours by use of in situ immunofluorescence staining. We are not aware of any previous study which has reported the expression of multiple glycolytic enzymes in ovarian tumours and certainly none that include a cohort of this size.

Analysis of histological subtype indicated higher expression of GLUT1 in HGSOC, the most frequently occurring form of epithelial ovarian cancer. Previous studies in small series of tumours have demonstrated increasing GLUT1 expression when comparing ovarian benign and borderline tumours to malignant ovarian adenocarcinomas and this transporter has been suggested as a potential marker of ovarian malignancy [52–54]. Our data is in line with a number of studies which have documented elevated GLUT1 expression in serous adenocarcinomas [53, 55–57]. Significantly higher GLUT1 expression was detected in advanced stage (III/IV) tumours compared to early stage (I/II) cancers. This is consistent with a previous report of increased GLUT1 expression being higher in advanced stage ovarian tumours [55]. GLUT1 has been proposed as a marker of adverse prognosis in ovarian cancer, however we did not observe an effect on survival in this cohort of patients [57]. Cantuaria et al. associated GLUT1 overexpression with poor disease free survival rate in 89 advanced stage ovarian carcinomas [58] while Semaan et al. demonstrated that high GLUT1 expression had a negative impact on the overall survival of 213 ovarian cancer patients [56]. Consistent with these reports, Cho et al. described a reverse statistically significant association among overall survival of 50 patients and high GLUT1 expression [57]. Enhanced tracer [F-18]-fluorodeoxyglucose (FDG) uptake, quantified by PET, has been shown to relate to increased GLUT1 expression in ovarian cancer and was related to increased cellular proliferation [59].

As for GLUT1, we observed that HKII was increased in HGSOC relative to non-HGSOC. The mitochondrial-bound HKII is the predominant isoform expressed in many tumours. Increased HKII expression has been noted in ovarian cancer for malignant tumours compared to benign and borderline tumours and increased HKII expression in serous carcinomas was found compared to non-serous tumours [60]. Suh et al. examined HKII expression by IHC in 111 ovarian tumours and documented that high HKII was correlated with chemoresistance and disease recurrence as well as decreased progression free survival [61].

The dependence of ovarian cancer cell growth on glucose was next assessed by investigating the effect of varying glucose concentration in culture. The mean physiological level of glucose in the plasma is approximately 5 mM, with a maximum concentration of 9 mM after eating and a minimum of 3 mM following physical exercise or moderate fasting [62]. Frequently the concentration of glucose in malignant tissues is significantly lower (up to 10 fold) than their normal counterparts in consequence of augmented glucose consumption and abnormal tumour microvasculature [63]. The ovarian cancer cell lines demonstrated differential ability to grow in the absence of glucose. TOV112D and OVCAR3 were both able to increase their cell number up to threefold in glucose depleted conditions while in contrast OVCAR5 and CAOV3 were unable to grow when glucose was not present in the culture medium (Fig. 5). For CAOV3 cells, a relatively high concentration equal to 0.4 mM was required for significant growth. Interestingly OVCAR5, TOV112D and CAOV3 cells reached a plateau of maximal growth at 1.6 mM glucose. In contrast, OVCAR3 cells demonstrated optimal growth when cultured in a low glucose environment of 0.4 mM. Glucose deprivation has been extensively associated with oxidative stress [64, 65]. Aykin-Burns et al. attributed the increased sensitivity of breast cancer cells to glucose withdrawal (and subsequently to glucose inhibition) compared to normal mammary epithelial cells, to the pro-oxidant status mediated by elevated ROS production [65]. In line with these findings Graham et al. also confirmed the association between the metabolic reconfiguration of tumours and increased sensitivity to glucose deprivation. They linked glucose depletion with elevated tyrosine kinase signalling and ROS mediated cell death [66].

In a previous report, we provided evidence that nine compounds targeting key components of the glycolytic pathway inhibited cancer cell proliferation in a concentration-dependent manner [40]. To explore this further, the effects of several inhibitors targeting key enzymes of the glycolytic pathway were investigated against paired chemosensitive/chemoresistant HGSOC cell line models. Recent evidence has associated drug resistance with an elevated dependency on the glycolytic phenotype however much less is known as to whether glycolysis inhibition could be exploited against resistant disease [67]. Targeting three major components of glycolysis proved effective in attenuating ovarian cancer cell proliferation in a concentration-dependent manner regardless of platinum sensitivity. The recently developed agents, IOM-1190, STF31 and 3PO were considerably more potent in inhibiting cancer cell proliferation compared to the more established oxamic acid that required concentrations in the millimolar concentration range (Table 1).

Currently, the administration of antitumour therapy generally involves combinatorial strategies of several therapeutic agents. Drug combinations aim to augment the therapeutic benefit, reduce the adverse effects and delay or ideally hinder resistance. Resistance to common chemotherapeutic agents has been associated with the deregulated reliance of tumours on the glycolytic pathway. It has been suggested that targeting the metabolic phenotype of tumours may enhance the efficacy of chemotherapy regimens and moreover resensitise tumour cells to treatment to which they had developed resistance [39, 40]. Possible proposed mechanisms predict glycolysis inhibition reducing cellular ATP levels and compromising the activation of resistance pathways or attenuating tumour growth promoting induction of apoptosis and hindering the adaptation to chemotherapeutic treatment [39, 40].

Platinum-based drugs are the most widely used agents for the treatment of ovarian cancer however platinum-refractory disease frequently develops and hence combinatorial treatments with other antitumour agents are currently under investigation, aiming to alleviate adverse effects and overcome resistance [68]. We observed that the PFKFB3 inhibitor 3PO significantly enhanced the cytotoxic effect of cisplatin against both platinum sensitive and platinum resistant ovarian cancer cells. This supports the view that combinatorial treatment of cisplatin with 3PO could reverse the platinum resistant phenotype and may be an effective strategy against platinum-resistant ovarian tumours. It should be noted that the concentrations of the two drugs that gave the lowest CI values are relatively low and potentially achievable in in vivo experiments. Paclitaxel (given 3-weekly) along with carboplatin is the other first line treatment for ovarian cancer. In addition, paclitaxel is also often used in a weekly schedule in platinum resistant disease. 3PO combined with paclitaxel produced synergistic anticancer action on ovarian cancer cells. Both PEA1 and PEA2 cell lines were very sensitive to this combination and the effectiveness of this combination especially for the resistant PEA2 line suggests that this combination might have in vivo potential.

To date a number of studies have revealed that certain compounds targeting the glycolytic metabolism of tumours might improve the therapeutic index of chemotherapeutic cytotoxic agents mainly through reduction of the ATP levels selectively in malignant cells [39, 40]. Similar to this study’s observations Liu et al. reported synergistic antitumour action between the GLUT1 inhibitor WZB117 and cisplatin or paclitaxel [69]. Another glucose transport inhibitor, the phytochemical Phloretin, has been shown to potentiate the cytotoxic effect of daunorubicin promoting apoptosis and also sensitised resistant leukaemia and colon cancer cells to the anthracycline exclusively under hypoxic conditions [70]. Nakano et al. documented that the HKII inhibitor 3BP enhanced the anticancer effects of daunorubicin and doxorubicin in leukaemia and myeloma cells both in vitro and in vivo. The glycolytic inhibitor diminished the cellular ATP levels which led to inactivation of the ATP-binding cassette transporters (ABC) therefore preventing the agent’s efflux from malignant cells [71].

Metformin is a biguanide widely used for the treatment of type 2 diabetes mellitus. The drug reduces insulin resistance and blood glucose levels through inhibition of mitochondrial respiratory chain complex 1 leading to reduced ATP production and subsequently provoking AMPK activation and mTOR inhibition [72, 73]. A considerable number of epidemiologic meta-analyses have associated metformin with a decreased incidence of several malignancies as well as with improved clinical outcome and reduced cancer-related mortality of diabetic cancer patients. Anti-proliferative action has been extensively demonstrated in preclinical studies in several types of cancer [71–76] and metformin is an attractive candidate for combinatorial cancer treatment. Experimentally, metformin enhanced the cytotoxic effect of several agents including cisplatin, paclitaxel and doxorubicin [72, 77, 78]. Metformin is currently being assessed in numerous clinical trials in various cancer types as chemoprevention, monotherapy or in combination with several chemotherapeutic agents [72–76, 79]. However, to date little attention has been paid to a possible interaction among glycolytic inhibitors and the antidiabetic drug. We previously reported a beneficial interaction between the glycolytic inhibitors STF31 and oxamic acid when combined with metformin in a triple negative breast cancer cell line model [40]. In the present study, we observed that metformin augmented STF31 and oxamic acid-induced cytotoxicity in both platinum sensitive and platinum resistant ovarian cancer cells. It was observed that while low concentrations of the antidiabetic drug and the glycolytic inhibitors had only marginal effects on the growth of ovarian cancer cell lines, in combination they induced a marked antitumour effect characterised by low synergistic CI values. This data extends our previous findings obtained in a breast cancer model [40] and provides further evidence that suggests that dual inhibition of the two energy pathways might be a promising antitumour therapeutic strategy for ovarian, as well as breast, cancer. Further research should now be undertaken to validate these promising in vitro pilot data and investigate their in vivo therapeutic potential.

## Conclusions

To the best of our knowledge this is the first study evaluating the expression of a series of glycolytic enzymes in a large cohort of ovarian tumours. We observed that HGSOC and advanced stage tumours frequently express higher levels of GLUT1 and HKII, the initial components of the pathway. Cell lines from HGSOC that are resistant to cytotoxic treatment retain comparable sensitivity to glycolytic inhibitors. Combination of glycolytic inhibitors with chemotherapy can produce significantly increased growth inhibition. This study supports further consideration of the use of glycolytic inhibitors for the treatment of ovarian cancer.

## Additional files

Additional file 1: **Table S1.** Number of ovarian cancer samples analysed by histology and stage. (DOCX 21 kb)

Additional file 2: TMA dataset. Mean AQUA expression values for GLUT1, LDHA, HKII and PKM2 in 380 ovarian cancer samples. Histology and stage are shown for individual tumours. (XLSX 77 kb)

Additional file 3: **Table S2.** Spearman correlation of the expression of four glycolytic enzymes in a cohort of 380 ovarian cancers. Spearman rho correlation values (top value) along with the respective adjusted *P* value (bottom value) of statistically significant correlations thresholded at FDR *P* < 0.01 are summarised. (DOCX 21 kb)

Additional file 4: **Figure S1.** Growth response curves of PE01 and PE04 ovarian cancer cells treated with combinations of glycolysis inhibitors with chemotherapy or metformin. **A.** 3PO with cisplatin. 3PO concentrations between 0.5-30μΜ alone (blue line) or combined with a constant concentration of cisplatin (red line) were evaluated. In green the effect of 0.5μΜ (PE01) or 1μΜ (PE04) cisplatin on cell viability is presented. **B.** 3PO with paclitaxel. 3PO concentrations between 0.5-30μΜ alone (blue line) or combined with a constant concentration of paclitaxel (red line) were evaluated. In green the effect of 2μΜ paclitaxel (both PE01 and PE04) on cell viability is presented. **C.** Oxamic acid with metformin. Concentration response curves of PE01 and PE04 ovarian cancer cells treated with oxamic acid concentrations between 1.56-100mΜ alone (blue line) or combined with 2 mM (PE01) or 0.5 mM (PE04) metformin (red line). In green the effect of 2 mM (PE01) or 0.5 mM (PE04) mM metformin on cell viability is presented. Cell viability was determined by an SRB assay after a 3-day treatment. Mean results of 6 replicates are reported and error bars represent standard deviations. Values are shown as a percentage of control. Asterisks indicate synergistic combination points with * CI value lower than 0.8 and ** CI value lower than 0.3. (PPTX 1444 kb)

## Abbreviations

2-DG: 2-Deoxy-D-glucose
3PO: 3-(3-Pyridinyl)-1-(4-pyridinyl)-2-propen-1-one
AQUA: Automated quantitative analysis
CI: Combination index
Cy3: Cyanine 3
DAPI: 4′,6-diamidino-2-phenylindole
DMEM: Dulbecco’s modified Eagle’s medium
EDTA: Ethylenediaminetetraacetic acid
FBS: Fetal bovine serum
FDR: False discovery rate
GLUT1: Glucose transporter 1
HGSOC: High grade serous ovarian cancer
HKII: Hexokinase II
IC_50_: Half maximal inhibitory concentration
IHC: Immunohistochemistry
IOM-1190: IOmet 1190
LDHA: Lactate dehydrogenase A
PBS: Phosphate buffered saline
PEP: Phosphoenolpyruvate
PET: Positron emission tomography
PFK1: Phosphofructokinase 1
PFKFB3: 6-phosphofructo-2-kinase/fructose-2,6-biphosphatase 3
PKM2: M2 isozyme of pyruvate kinase
ROS: Reactive oxygen species
SRB: Sulphorhodamine B
STF31: 4-[[[[4-(1,1-Dimethylethyl)phenyl]sulfonyl] amino] methyl]-N-3-pyridinylbenzamide
STR: Short tandem repeat profiling
TCA: Trichloroacetic acid
TMA: Tissue microarray
