# Supplementary material for: Expression of glycolytic enzymes in ovarian cancers and evaluation of the glycolytic pathway as a strategy for ovarian cancer treatment
Source: BMC Cancer. 2018 Jun 5;18:636. doi: 10.1186/s12885-018-4521-4 (PMC5987622; doi:10.1186/s12885-018-4521-4)
Supplement: Supplementary file 1 — Table S1. Number of ovarian cancer samples analysed by histology and stage. (DOCX 21 kb) [file 12885_2018_4521_MOESM1_ESM.docx]

**Additional file 1: Table S1.** Number of ovarian cancer samples analysed by histology and stage.

| **Histology** | **HGSOC** | **Endometrioid** | **Clear cell** | **Mucinous** | **LGSOC** |
| --- | --- | --- | --- | --- | --- |
|  | 282 | 55 | 25 | 10 | 8 |
| **Stage** | **I** | **II** | **III** | **IV** | **Unknown** |
|  | 39 | 43 | 219 | 66 | 13 |

HGSOC refers to high grade serous ovarian cancer, while LGSOC refers to low grade serous ovarian cancer.
