# Supplementary material for: Expression of glycolytic enzymes in ovarian cancers and evaluation of the glycolytic pathway as a strategy for ovarian cancer treatment
Source: BMC Cancer. 2018 Jun 5;18:636. doi: 10.1186/s12885-018-4521-4 (PMC5987622; doi:10.1186/s12885-018-4521-4)
Supplement: Supplementary file 3 — Table S2. Spearman correlation of the expression of four glycolytic enzymes in a cohort of 380 ovarian cancers. Spearman rho correlation values (top value) along with the respective adjusted P value (bottom value) of statistically significant correlations thresholded at FDR P < 0.01 are summarised. (DOCX 21 kb) [file 12885_2018_4521_MOESM3_ESM.docx]

**Additional file3: Table S2:** Spearman correlation of the expression of four glycolytic enzymes in a cohort of 380 ovarian cancers. Spearman rho correlation values (top value) along with the respective adjusted P value (bottom value) of statistically significant correlations thresholded at FDR P<0.01 are summarised.

| Nonparametric  Correlation | **GLUT1** | **HKII** | **PKM2** | **LDHA** |
| --- | --- | --- | --- | --- |
| **GLUT1** | * | 0.202  0.007 | 0.269  0.0002 | 0.234  0.002 |
| **HKII** | 0.202  0.007 | * | 0.340  0.000002 | - |
| **PKM2** | 0.269  0.0002 | 0.340  0.0000025 | * | 0.456  5.02e^-12^ |
| **LDHA** | 0.234  0.002 | - | 0.456  5.02e^-12^ | * |
