# Supplementary material for: Expression of glycolytic enzymes in ovarian cancers and evaluation of the glycolytic pathway as a strategy for ovarian cancer treatment
Source: BMC Cancer. 2018 Jun 5;18:636. doi: 10.1186/s12885-018-4521-4 (PMC5987622; doi:10.1186/s12885-018-4521-4)
Supplement: Supplementary file 4 — Figure S1. Growth response curves of PE01 and PE04 ovarian cancer cells treated with combinations of glycolysis inhibitors with chemotherapy or metformin. A. 3PO with cisplatin. 3PO concentrations between 0.5-30μΜ alone (blue line) or combined with a constant concentration of cisplatin (red line) were evaluated. In green the effect of 0.5μΜ (PE01) or 1μΜ (PE04) cisplatin on cell viability is presented. B. 3PO with paclitaxel. 3PO concentrations between 0.5-30μΜ alone (blue line) or combined with a constant concentration of paclitaxel (red line) were evaluated. In green the effect of 2μΜ paclitaxel (both PE01 and PE04) on cell viability is presented. C. Oxamic acid with metformin. Concentration response curves of PE01 and PE04 ovarian cancer cells treated with oxamic acid concentrations between 1.56-100mΜ alone (blue line) or combined with 2 mM (PE01) or 0.5 mM (PE04) metformin (red line). In green the effect of 2 mM (PE01) or 0.5 mM (PE04) mM metformin on cell viability is presented. Cell viability was determined by an SRB assay after a 3-day treatment. Mean results of 6 replicates are reported and error bars represent standard deviations. Values are shown as a percentage of control. Asterisks indicate synergistic combination points with * CI value lower than 0.8 and ** CI value lower than 0.3. (PPTX 1444 kb) [file 12885_2018_4521_MOESM4_ESM.pptx]

## Slide 1
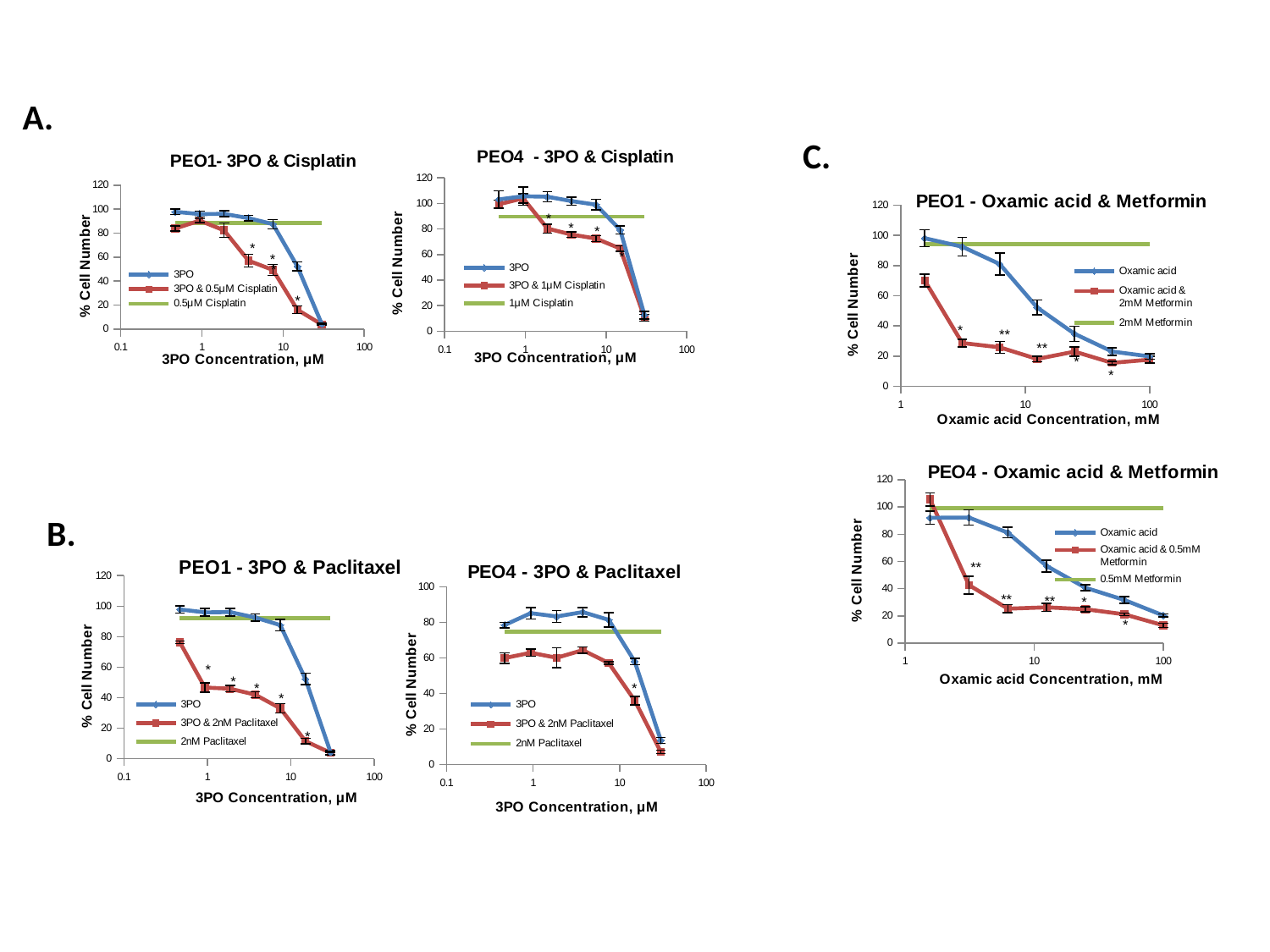

A.
### Chart: PEO4 - 3PO & Cisplatin
| Category | 3PO | 3PO & 1μM Cisplatin | 1μM Cisplatin |
|---|---|---|---|C.
### Chart: PEO1- 3PO & Cisplatin
| Category | 3PO | 3PO & 0.5μM Cisplatin | 0.5μΜ Cisplatin |
|---|---|---|---|
### Chart: PEO1 - Oxamic acid & Metformin
| Category | Oxamic acid | Oxamic acid & 2mM Metformin | 2mM Metformin |
|---|---|---|---|*
*
*
*
*
*
*
*
**
**
*
*
### Chart: PEO4 - Oxamic acid & Metformin
| Category | Oxamic acid | Oxamic acid & 0.5mM Metformin | 0.5mM Metformin |
|---|---|---|---|B.
### Chart: PEO1 - 3PO & Paclitaxel
| Category | 3PO | 3PO & 2nM Paclitaxel | 2nM Paclitaxel |
|---|---|---|---|**
### Chart: PEO4 - 3PO & Paclitaxel
| Category | 3PO | 3PO & 2nM Paclitaxel | 2nM Paclitaxel |
|---|---|---|---|**
**
*
*
*
*
*
*
